# Loss of *Xenopus tropicalis* EMSY causes impairment of gastrulation and upregulation of *p53*

**DOI:** 10.1016/j.nbt.2010.10.010

**Published:** 2011-07

**Authors:** Amer A. Rana, Stephen J. Roper, Elizabeth A. Palmer, James C. Smith

**Affiliations:** 1 Wellcome Trust/Cancer Research UK Gurdon Institute, Department of Zoology, University of Cambridge, Cambridge, UK; 2 Division of Respiratory Medicine, Department of Medicine, Box 157, 5th Floor, University of Cambridge, Addenbrooke's Hospital, Hills Road, Cambridge CB2 0QQ, UK; 3 The Babraham Institute, Cambridge CB22 3AT, UK; 4 Protein Technology Group, Babraham Bioscience Technologies, Babraham Research Campus, Cambridge CB22 3AT, UK; 5 MRC National Institute for Medical Research, The Ridgeway, Mill Hill, London NW7 1AA, UK

## Abstract

EMSY interacts directly with BRCA2 and links the BRCA2 pathway to sporadic breast and ovarian cancer. It also interacts with BS69 and HP1b, both of which are involved in chromatin remodelling, and with NIF-1 and DBC-1 in the regulation of nuclear receptor-mediated transcription. Here we investigate the function of EMSY during amphibian development, and in doing so provide the first loss-of-function analysis of this protein. Injection of *Xenopus tropicalis* embryos with antisense morpholino oligonucleotides targeting XtEMSY impairs gastrulation movements, disrupts dorsal structures, and kills embryos by tailbud stages. Consistent with these observations, regional markers such as *Xbra*, *Chd*, *Gsc*, *Shh*, *Sox3* and *Sox17* are downregulated. In contrast to these regional markers, expression of *p53* is upregulated in such embryos, and at later stages *Bax* expression is elevated and apoptotic cells can be detected. Our results demonstrate that EMSY has an essential role in development and they provide an *in vivo* loss-of-function model that might be used to explore the biochemical functions of this protein in more detail.

## Introduction

The EMSY protein is upregulated in breast and ovarian cancers [1]. The N-terminal region of the protein interacts with the transactivation domain of BRCA2 and with the chromatin modelling-associated proteins BS69 and HP1b [1]. This region defines an evolutionarily conserved ENT (EMSY N-Terminus) domain that is represented in plants as well as in animals, although EMSY is the only known ENT-containing protein in the human proteome [1,2]. More recently EMSY has been shown to participate in a complex with NIF-1 and DBC-1 in the regulation of nuclear receptor-mediated transcription [3]. Finally, EMSY co-localises at γ-H2AX foci following radiation-induced double-strand DNA breaks in mouse embryonic fibroblasts, suggesting that it may also have a role in DNA damage repair [1]. Indeed, over-expression of a truncated from of EMSY results in chromosomal instability, although this construct was expressed at levels ten times higher than those in naturally occurring tumours [4]. Thus, in adult cells EMSY is implicated in a variety of cellular processes including gene transcription, chromatin remodelling, and DNA repair.

In this study, in an effort to gain further insights into the functions of EMSY, we address its role during early development, and in so doing provide the first loss-of-function analysis of EMSY. The *Xenopus tropicalis* genome contains a gene that is highly homologous to mammalian *EMSY* (*XtEMSY*). A ‘short’ form of *XtEMSY* is expressed throughout early development and is co-expressed with mRNAs encoding interacting proteins such as BRCA2, BS69 and HP1b. Use of antisense morpholino oligonucleotides directed against *XtEMSY* disrupts gastrulation and causes a downregulation of genes including *Gsc* and *Shh*. At later stages we observe an upregulation of *p53*, which may underlie the elevated levels of apoptosis we observe and, through its ability to interact with the TGF-β signal transduction pathway (5), may exacerbate the defects in embryonic patterning.

## Materials and methods

### Embryos and *in vitro* fertilization

Embryo generation and manipulation were carried out as described [5].

### MO design and microinjection

Antisense morpholino oligonucleotides (MOs) were obtained from GeneTools. Embryos were injected with 15 ng antisense morpholino oligonucleotides at a concentration of 10 ng/nl in water. MOs had the following sequences. XtEMSY MO1: 5′-CCACACCACCGGCATCCTGGCCTCT-3′; XtEMSY mMO1: 5′-CCACACCACCGGCATCCTGGCCTCT-3′, XtEMSY MO2: 5′-TGGCCTCTCCTCCACAGAGCGCCCT-3′; XtEMSY mMO2: 5′-TGGCCTCTCCTCCACAGAGCGCCCT-3′; Xtp53MO: 5′-GCCGGTCTCAGAAGAAGGTCCCATG-3′.

### RT-PCR

RNA extraction was carried out using TRIZOL reagent (Invitrogen) according to the manufacturer's instructions (except that RNA was LiCl precipitated a second time at the end of the protocol). cDNA synthesis was carried out using SuperscriptII (Invitrogen) and random hexamer primers. The following primers were used in RT-PCR: *XtEMSY* forward: 5′-GCCAGGATGCCGGTGGTG-3′; reverse: 5′-GCGTTTATTCCAGGGATCCTCTG-3′. *EF1α* forward: 5′-TGGACACGTAGATTCTGG-3′; reverse: 5′-CAGCAACAATCAGGACAG-3′.

### *In situ* hybridisation

Whole mount *in situ* hybridisation was carried out as described [6], using DIG-labelled probes and BM purple (Roche) as substrate. Full-length probes were generated from the following cDNAs, in pCS107, picked from the Gurdon Institute *X. tropicalis* cDNA collection. They were digested with EcoRI and transcribed with T7 RNA polymerase*. Xbra*: Tgas071i19; *Chd*: Tgas143n13; *EMSY*: Tgas029a21; *Gsc*: Tneu077f20; *p53*: Tneu034a23; *Rad51*: Tegg126n13; *Shh*: Tneu023n04; *Sox3*: Tgas079n14; *Sox17a*: Tgas079e22.

### TUNEL staining

TUNEL staining was carried out as described [7].

### Real-time RT-PCR

Real-time RT-PCR was performed using a Roche Lightcycler 480. The following primer pairs were used: *ODC* forward: 5′-GCCATCGTGAAGACTCTCTCCC-3′; reverse: 5′-TTCGGGTGATTCCTTGCCAC-3′. *p53* forward: 5′-AAACTTTGCGGAGTTTTCAGAG-3; reverse: 5′-GGTGGAGTATGTGCAGGTAACA-3′. *Bax* forward: 5′-GAGCCTTGGTGCTGCAGGGG-3′; reverse: 5′-GGAGCCTGGGAATAGCGCCC-3′. *Xbra* forward: 5′-ATCAAACACAACCCCTTTGC-3′; reverse: 5′-CGAGCGGTGGTTTCTTAGAG-3′. *Chd* forward: 5′-AACTGCCAGGACTCATGGATG-3′; reverse: 5′-GGCAGGATTTAGAGTTGCTTC-3′. *Gsc* forward: 5′-GTTTTCAGCCAGGAGAGAGAGA 3′; reverse: 5′-ATGTTGTCAATGCTGAACATGC-3′. *Shh* forward: 5′-AGCCTTTGATGTAATTGGCTTC-3′; reverse: 5′-AATCTTTCCTTCGTATCGACCA-3′.

## Results

### Identification of *Xenopus* EMSY

To identify the *Xenopus* orthologue of human *EMSY* we performed sequence searches with the full-length human EMSY sequence using the University of California Santa Cruz (UCSC) genome browser (http://genome.ucsc.edu/index.html?org=X.+tropicalis&db=xenTro2&hgsid=129491895), the *X. tropicalis* full-length EST database (http://informatics.gurdon.cam.ac.uk/online/xt-fl-db.html) [8], and EST databases at NCBI (http://www.ncbi.nlm.nih.gov) [9,10]. We thus identified an *X. tropicalis* locus that is orthologous to human *EMSY* on scaffold_609 between positions 307237 and 325312. Our analysis of the *X. tropicalis EMSY* locus indicates that it consists of 22 exons, the first 21 of which are predicted to generate a protein of 1291 amino acids which is 78% identical to its 1322 amino acid human orthologue (Fig. 1a). Our *X. tropicalis* EST database searches, however, identified only a transcript that corresponds to exons 1–7 and exon 22, and which encodes a protein of 269 amino acids (Fig. 1b). This protein is 94% identical to amino acids 1–255 of human EMSY and it contains the ENT domain, which interacts with BRCA2, as well as the HP1β and BS69 binding sites, making it possible that the protein is involved in transcriptional regulation, chromatin modification and DNA repair. Our PCR analysis (below) confirms that this transcript is expressed in the early *Xenopus* embryo, and we note that an EST encoding a 299 amino acid protein is also present in a cow EST library.

### Expression of *Xenopus tropicalis* EMSY

Although we have only been able to identify a short form of *XtEMSY* from database searches, RT-PCR analysis reveals that a full-length version is expressed in adult tissues such as testis and brain (Fig. 2a). The short form is expressed in the embryo at least from the 2-cell stage to tadpole stage 40, and there is a significant upregulation at early gastrula stage 10 (Fig. 2b). Whole mount *in situ* hybridisation experiments, using a probe complementary to the short form of Xt*EMSY*, showed that at gastrula stages expression is widespread through the ectoderm and that at later stages it is strongest in anterior dorsal tissues such as anterior neural epithelium, head, neural tube and somites (Fig. 3a–h).

### XtEMSY mRNA is co-expressed with RNA encoding potential interacting proteins

EMSY interacts with various proteins involved in transcriptional regulation, chromatin modification and DNA repair, including BRCA2, BS69, ETS-2 and HP1β [1]. Transcripts encoding the *X. tropicalis* orthologues of these proteins proved to have similar expression patterns to *XtEMSY* during early development (Fig. 3i–x), defining them as members of a synexpression group [5]. This observation is consistent with the possibility that XtEMSY interacts with the same proteins as its mammalian counterpart, and that it plays the same roles as the mammalian protein.

### Loss of XtEMSY function causes defects in gastrulation

Although over-expression of a truncated form of human EMSY in 184-hTert cells causes chromosomal abnormalities [4], we saw no effect of over-expression of up to 1.2 ng wild type *XtEMSY* RNA (the short form) in *X. tropicalis* embryos (data not shown). This apparent discrepancy may derive from differences between truncated human EMSY and the short form of XtEMSY, from differences between *in vitro* and *in vivo* systems, or from differences between adult and embryonic cells.

In an effort to inhibit XtEMSY function, we turned to antisense morpholino oligonucleotides (MOs) [5,11,12]. We used two independent MOs designed to inhibit translation of XtEMSY (XtEMSY MO1 and XtEMSY MO2), and as controls we made use of the GeneTools control MO as well as MOs carrying five base mismatches compared with their specific versions (XtEMSY mMO1 and XtEMSY mMO2). Both specific MOs yielded a phenotype that was characterised by a delay in the onset of gastrulation and then by defects in head and axial development (Fig. 4c–f), followed by death at tailbud stages (data not shown). None of the three control MOs caused a detectable disruption of development (Fig. 4a,b,j–l,p–t). Because both MOs directed against XtEMSY yielded the same phenotype, we henceforth present data using only XtEMSY MO1 unless otherwise stated.

Our attempts to rescue the XtEMSY MO phenotype by injection of mRNA encoding the short form of XtEMSY did not succeed (data not shown). It is possible that injected mRNA does not spread rapidly enough throughout the embryo, or that XtEMSY protein is unstable and does not persist to the appropriate stages.

### Analysis of early patterning markers during gastrula and tailbud stage

Antisense MOs directed against XtEMSY cause a delay in gastrulation followed by a disruption of neurulation and of further development. To understand the molecular basis of this phenotype, we used whole mount *in situ* hybridisation to examine the expression of genes including *Xbra* (expressed in mesoderm), *Chordin* (*Chd*: chordamesoderm), *Goosecoid* (*Gsc*: dorsal mesendoderm), *Sonic hedgehog* (*Shh*: chordamesoderm), *Sox3* (neurectoderm) and *Sox17a* (endoderm) (Fig. 5a–x). Expression of all six genes was reduced in embryos injected with MOs targeting XtEMSY, although their spatial expression patterns appeared normal. Of the genes examined, *Chd* and *Shh* were the most significantly downregulated, suggesting that notochord formation is impaired, consistent with the impairment of axial development observed above.

These results were confirmed by real-time RT-PCR, which revealed that embryos injected with XtEMSY MO1 have reduced levels of *Xbra*, *Chordin*, *Goosecoid* and *Sonic hedgehog* (Fig. 5y).

### Downregulation of EMSY causes an elevation of p53 expression

EMSY was discovered in a screen for BRCA2 interactors and it links the BRCA2 pathway to sporadic breast and ovarian cancer [1]. It also localises to γ-H2AX foci following radiation-induced double-strand DNA breaks. Bearing these observations in mind, we used whole mount *in situ* hybridisation to examine the expression of cell cycle and DNA damage regulators in embryos injected with XtEMSY antisense morpholino oligonucleotides and noted that while *cdc1*, *cdc2*, *Rb1*, *Rb2* and *Rad51* remained unchanged, *p53* was upregulated in such embryos during gastrula and neurula stages (Fig. 6a,b and data not shown). This conclusion was confirmed by real-time RT-PCR, although a substantial upregulation of *p53* was not noted until after stage 18 (Fig. 6c). p53 interacts with the Smad signal transduction pathway downstream of TGF-β family members; its over-expression in isolated animal pole regions activates mesodermal and mesendodermal genes such as *Xhox3* and *Mix.1/2*, while loss of p53 in the intact embryo inhibits mesoderm formation [13–15]. Together, these observations make it unlikely that the loss of mesodermal markers observed in embryos injected with XtEMSY antisense morpholino oligonucleotides (Fig. 5) is a direct consequence of the modulation of p53, and indeed we find that we cannot rescue the effects of XtEMSY MO1 by means of an antisense morpholino oligonucleotide targeted against *X. tropicalis* p53 (data not shown).

### Upregulation of p53 in embryos lacking XtEMSY is followed by upregulation of Bax and by apoptosis

Although p53 upregulation is unlikely to influence directly the decrease in expression of genes such as *Gsc* and *Shh*, we note that expression of the apoptosis associated gene *Bax* [16] is also upregulated in embryos injected with XtEMSY MO1 (Fig. 6c). Consistent with this observation, TUNEL staining of embryos injected with XtEMSY MO1 reveals that apoptotic cells can be detected from late neurula to tailbud stages, with a particular concentration in dorsal tissues (Fig. 7).

## Discussion

*XtEMSY*, the orthologue of human *EMSY*, is expressed as a short isoform during early development of *X. tropicalis* and is co-expressed with genes encoding proteins that interact with its human homologue, suggesting that XtEMSY might be involved in transcriptional regulation, chromatin remodelling and DNA repair.

Loss of EMSY function in *X. tropicalis* disrupts gastrulation and axial development. Expression of the regional markers *Xbra*, *Chd*, *Gsc*, *Shh*, *Sox3* and *Sox17a* is reduced at gastrula and tailbud stages, although their spatial expression patterns are little affected. In contrast, *p53* is upregulated during gastrula stages, as is *Bax* at neurula stages. The upregulation of these genes may underlie the apoptosis observed in XtEMSY knockdown embryos [16]. It is unlikely, however, that the upregulation of *p53* or *Bax* disrupts gastrulation or causes the loss of regional markers, because expression of the two genes increases only after these phenotypes are detectable, and injection of an antisense morpholino oligonucleotide directed against *X. tropicalis* p53 cannot rescue the embryos. Furthermore, upregulation of *p53* activates the expression of mesodermal markers rather than inhibiting their transcription [14,15].

Using our current study as a platform, in future work we will aim to investigate *Xt*EMSY function in the spatio-temporal control of gene transcription including epigenetic regulation and epigenomic remodelling (determining cell fate in this case) *versus* its role in replication, similar to Polycomb protein group protein-containing complexes, which modulate whether chromatin is in a closed or open state (in conjunction with Top2) independently from their role in replication (in conjunction with Top1). This type of work is usually only addressed in studies based on cell lines, addressing in this case ‘stem cellness’, transit amplification and differentiation. However through our initial characterisation we have the first opportunity to take this type of analysis *in vivo.*

Finally this work also demonstrates the usefulness of the *Xenopus* morphant system as a technique for discovering the potential functions of genes found in mammals for which the loss-of-function models yield no obvious phenotype.

## Figures and Tables

**Figure:**
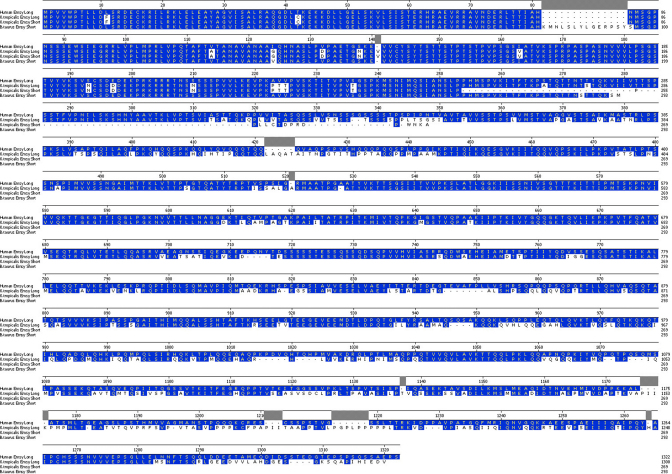


**Figure 1:**
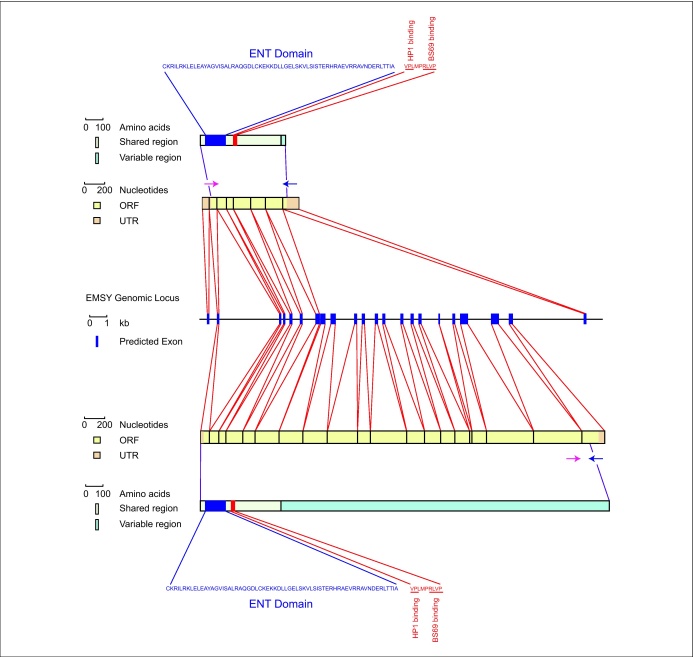
Genomic structure of the *XtEMSY* locus and of XtEMSY isoforms. The *XtEMSY* locus comprises 22 exons, of which the first 21 encode a protein of 1291 amino acids. A short transcript comprising exons 1–7 and exon 22, encoding a protein of 269 amino acids, was detected in early embryos of *Xenopus tropicalis*. ESTs representing this transcript were found in egg, gastrula and tadpole cDNA libraries: AL870737.2 (egg); AL871852.2 (egg); AL652745.2 (gastrula); and CX371164.1 (tadpole). The short and the long forms of *XtEMSY* both contain the ENT domain and putative HP1β and BS69 binding sites.

**Figure 2:**
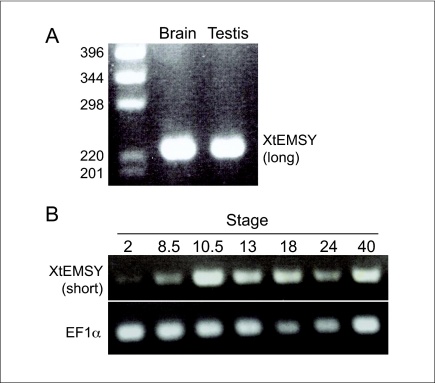
Expression of *XtEMSY* during development. RT-PCR confirms that the long form of *XtEMSY* is expressed in adult brain and testis **(a)**, and that the short form is expressed at the 2-cell stage and at embryonic stages 8.5, 10.5, 13, 18, 24 and 40 **(b)**. Primers specific for *EF1α* were used as a control.

**Figure 3:**
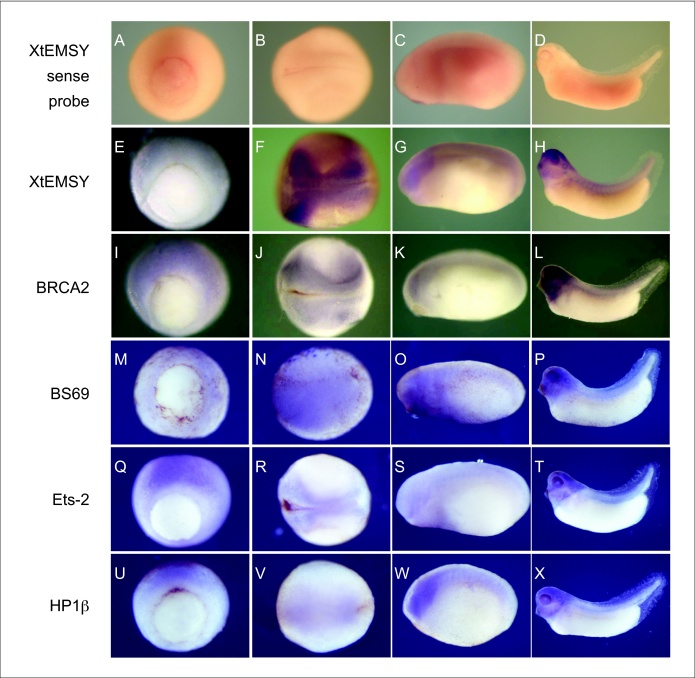
Analysis of the expression of *XtEMSY* and of some of its putative interactors. Gastrula stages are shown in panels **(a, e, i, m, q, u)**, neurula stages in panels **(b, f, j, n, r, v)**, tailbud stages in panels **(c, g, k, o, s, w)** and tadpole stages in panels **(d, h, l, p, t, x)**. (a–d) Use of an *XtEMSY* sense probe shows no staining. (e–h) *XtEMSY* is expressed throughout the ectoderm at the gastrula stage (e) and is then expressed most strongly in the neural plate and dorsal regions at the neurula stage (f). By tailbud stages *XtEMSY* is expressed most strongly in the head and along the dorsal axis (g). At tadpole stage *XtEMSY* staining is observed in the entire head region, including the brain, eye, otic vesicle and branchial arches and along the dorsal axis in the neural tube and somites (h). The expression patterns of *BRCA2* (i–l), *BS69* (m–p), *Ets-2* (q–t) and *HP1β* (u–x) resemble those of *XtEMSY*.

**Figure 4:**
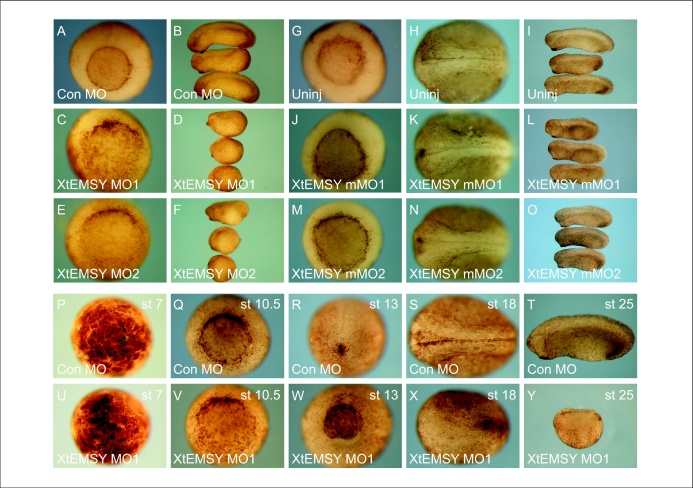
Loss of XtEMSY function causes defects in gastrulation and neurulation. Embryos injected with GeneTools control MO develop normally **(a and b)**, whereas those injected with XtEMSY MO1 **(c and d)** or XtEMSY MO2 **(g and f)** fail to gastrulate normally and form a truncated axis. **(g–o)** Embryos injected with antisense morpholino oligonucleotides carrying five bases mismatches do not perturb development. **(g–i)** Uninjected embryos at gastrula, neurula and tailbud stages. **(j–l)** Embryos at the same stages injected with XtEMSY mMO1, and **(m–o)** show embryos injected with XtEMSY mMO2. **(p–y)** A time course experiment shows that in comparison with controls **(p–t)** embryos injected with XtEMSY MO1 **(u–y)** first show abnormalities at the early gastrula stage (compare **q** and **v**).

**Figure 5:**
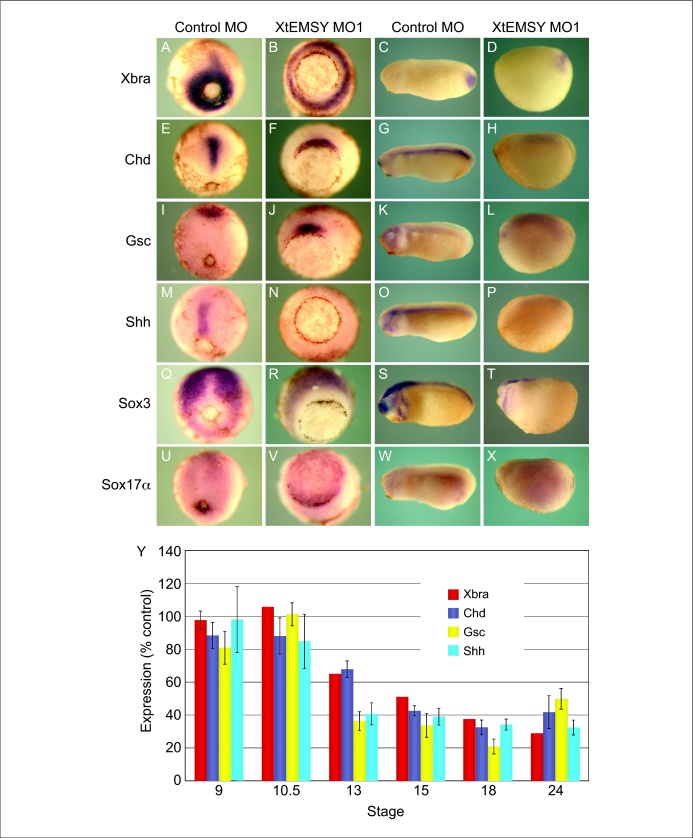
Expression of various regional markers is down regulated in embryos injected with XtEMSY MO1. **(a–x)** The expression patterns of the indicated genes at gastrula and tailbud stages in control embryos and embryos injected with XtEMSY MO1. Note the impairment of gastrulation and the downregulation of, particularly, Shh **(m–p)**. The spatial expression patterns of the analysed genes are only slightly affected. **(y)** Real-time RT-PCR shows that expression of *Xbra* and *Chd* is slightly decreased by loss of XtEMSY function, while expression of *Gsc* and *Shh* is down regulated more strongly.

**Figure 6:**
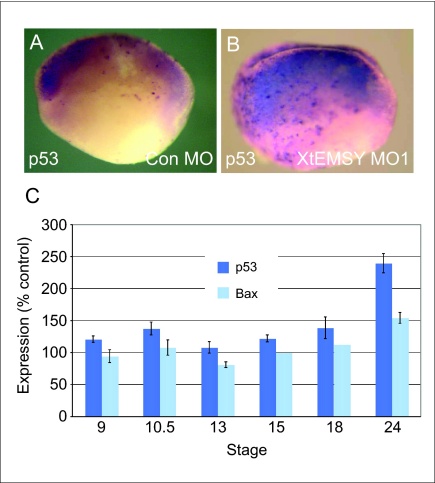
Loss of XtEMSY function causes upregulation of *p53*. **(a and b)***In situ* hybridisation analysis at the late neurula stage reveals that *p53* is upregulated in embryos injected with XtEMSY MO1 (b) compared with controls. **(c)** Temporal RT-PCR analysis of p53 and Bax expression in embryos injected with XtEMSY MO1. Expression levels are normalised to those of ornithine decarboxylase and levels in control MO injected embryos are defined as 100%. Note that expression of *p53* is elevated between stages 18 and 24 (dark blue), while *Bax* is upregulated slightly later than this.

**Figure 7:**
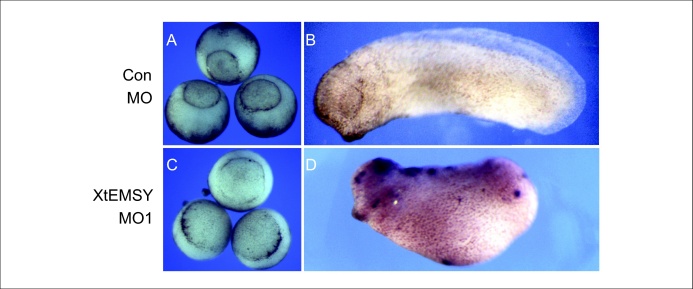
Apoptosis in embryos injected with XtEMSY MO1. Control embryos **(a and b)** or embryos injected with XtEMSY MO1 **(c and d)** were subjected to TUNEL staining. No apoptosis was detected at stage 11 (a and b), but apoptotic cells were present by tailbud stages in dorsal regions (c and d).
